# Ammonia Retention in Biowaste via Low-Temperature-Plasma-Synthesized Nitrogen Oxyacids: Toward Sustainable Upcycling of Animal Waste

**DOI:** 10.1021/acssuschemeng.3c06423

**Published:** 2024-02-05

**Authors:** Victor V. Miller, Douglas S. Clark, Ali Mesbah

**Affiliations:** Department of Chemical and Biomolecular Engineering, University of California, Berkeley, California 94720, United States

**Keywords:** low-temperature plasma, animal waste processing, manure upcycling, nitrogen fixation, green fertilizer

## Abstract

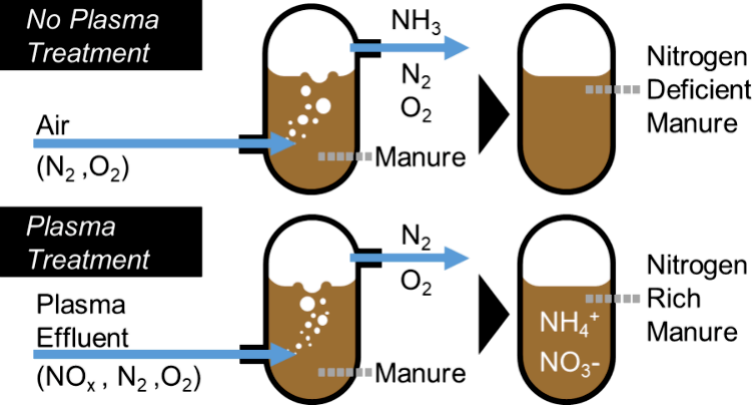

Sustainable fertilizer production is a pressing challenge due to a growing human population. The manufacture of synthetic nitrogen fertilizer involves intensive emissions of greenhouse gases. The synthetic nitrogen that ends up in biowaste such as animal waste perturbs the nitrogen cycle through significant nitrogen losses in the form of ammonia volatilization, a major human health and environmental hazard. Low-temperature air-plasma treatment of animal waste holds promise for sustainable fertilizer production on farmlands by enabling nitrogen fixation via ionization, forming nitrogen oxyacids. Although the formation of nitrogen oxyacids in plasma treatment of water is well-established, the extent of nitrogen oxyanion enrichment in animal waste and its downstream effects on acidifying the waste remain elusive because many compounds found in complex biowaste media may interfere with absorbed NO_*x*_ species. This work aims to establish that plasma treatment of dairy manure can suppress ammonia loss by volatilization via acidification of animal waste while enriching the waste in total nitrogen due to nitrogen retained in ammonia as well as adding nitrogen oxyacids by reacting NO_*x*_ with the aqueous slurry. To this end, air-plasma effluent containing NO_*x*_ is bubbled through dairy manure, which is then analyzed for changes in the nitrogen oxyanion content and pH. Increasing the plasma treatment time results in more acidic manure, reduced ammonium content in the downstream acid trap, and increased nitrogen oxyanion content, where the yield of nitrogen oxyanion from absorbed NO_*x*_ species is approximately 100%. Increased plasma treatment also led to an increase in the total Kjeldahl nitrogen and the total nitrogen. These results indicate that plasma treatment of animal waste can significantly suppress ammonia pollution from animal husbandry facilities such as dairy farms while upcycling animal waste as a rich organic source of nitrogen.

## Abstract 2

This work demonstrates that plasma-based chemistry via renewable electricity can upcycle biowaste into enriched organic fertilizer and reduce ammonia pollution.

## Introduction

There is an urgent need to produce food and, hence, fertilizer in an environmentally sustainable manner. This requirement is driven by human population growth, which is projected to increase from approximately 8 billion at the end of 2022 to approximately 10 billion in 2050.^1^ Historic population growth in the past decades has been enabled by inexpensive fixed-nitrogen fertilizer generated via the Haber-Bosch (HB) process, which is expected to sustain the projected population growth.^2^ However, the HB process leads to enormous greenhouse gas emissions, exceeding 300 million metric tonnes of CO_2_.^3−5^ Synthetic fertilizer is also responsible for large N_2_O emissions, which have 300 times the warming potential of CO_2_.^6^ Moreover, significant quantities of ammonia evaporate from agricultural fields and rotting biomass. Up to 2% of ammonia introduced to the ecosystem is transformed into N_2_O.^7^ Much of this nitrogen is sourced from synthetic nitrogen produced by the HB process. These large flows of reactive nitrogen pollute air and water, are associated with increased cancer rates, can form algal blooms, and/or cause aquatic deadzones.^8^ This reactive nitrogen perturbs the global nitrogen cycle to the extent that it is said to be approaching a “planetary boundary” and threatening global ecosystems.^9^ The U.S. National Academy of Engineering recognized this threatening situation as one of the 14 major grand challenges for engineering in the 21st century, labeling it “Managing the Nitrogen Cycle.”^10^

Perturbation of the global nitrogen cycle starts mostly at the farm level. In 2005, the California Nitrogen Assessment estimated that ammonia nitrogen losses from animal husbandry facilities alone were over 20% of the synthetic nitrogen applied to farmland,^11^ while 20–70% of nitrogen in manure applied to farmland is lost.^12^ Herein lies an opportunity to improve efficiency. This lost ammonia can be retained by acidifying the ammonia to involatile ammonium. The pH of manure is in the range of 7–9;^13−15^ thus, in principle, it is possible to prevent the loss of volatile ammonia by acidifying the animal waste,^16^ i.e.
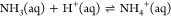
1This reaction is reversible, and the fraction of combined ammonia and ammonium that exists in either state is pH-dependent. The p*K*_a_ of ammonia at 25 °C is 9.25,^17^ such that a significant amount of ammonia is un-ionized in manure without some type of acidification treatment. It has been postulated that treating animal waste with low-temperature plasma (LTP), specifically air-plasma effluent, can generate nitric or nitrous acid in the waste to retain the otherwise lost ammonia.^16,18^ The addition of nitric or nitrous acid can further increase the nitrogen content of animal waste. Upcycling animal waste in this way is desirable because it would enable green fertilizer production without CO_2_ production and would also reduce perturbation of the global nitrogen cycle. It has been shown that gas-phase plasma nitrogen fixation and subsequent transformation to nitrogen oxyacids in aqueous systems can be performed at atmospheric pressure.^19^ It is yet to be demonstrated, however, that this can acidify animal waste, or any other forms of biowaste, and enrich the nitrogen in biowaste by adding nitrogen oxyanions.

To provide context for the contribution of this work, we first give a brief overview of gas- and liquid-phase chemistry for air-plasma nitrogen fixation in water. LTP treatment can fix nitrogen in the form of NO_*x*_ from air via the nonthermal Zeldovich mechanism^19−21^
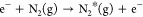
2
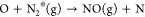
3
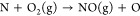
4Equation 2 yields vibrationally excited dinitrogen (N_2_*) via electron impact. It is then much easier to overcome the dinitrogen triple bond via N_2_* reacting with atomic oxygen (eq 3). The overall reaction for eqs 2–4 is
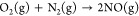
5The synthesis of NO_*x*_ can also proceed thermally,^21^ while NO oxidizes in air, i.e.
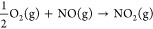
6NO_*x*_ species can then react with each other to create other forms of nitrogen oxides and can react with water at the air-water interface to form nitrous acid and nitric acid^19,22^
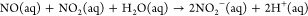
7
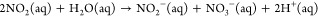
8
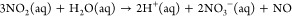
9
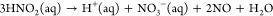
10Note that nitrous acid and its anion, nitrite, can be oxidized to nitric acid and nitrate, respectively. Other reactive species observed in atmospheric pressure air plasmas include ozone, hydroxyl radicals, and hydrogen peroxide. Additional aqueous reactions for the creation of nitrogen oxyacids are possible, as are other reactions between nitrogen oxyacids and between nitrogen oxyacids and gas-phase nitrogen species; see refs (16), (18), (19), and (21)–^25^ for detailed descriptions of the gas- and aqueous-phase chemistry.

The reactions in eqs 2–10 demonstrate how plasma treatment of an aqueous solution can produce nitrogen oxyanions and reduce the pH of the solution. In particular, the reactions in eqs 7–10 provide the acidification needed to ionize ammonia to ammonium, as given in eq 1. However, while the occurrence of reactions (eqs 7–10) in water has been established,^16,18,19,22−25^ the extent to which these reactions can occur in biowaste has not been previously investigated. The intermediate species in these reactions (e.g., NO_*x*_) are potent oxidizers. Additionally, the end products, namely, nitrite and nitrate, are oxidizers and reactive.^26−29^ Hence, these reactive intermediates and products could react with many compounds in complex biowaste media.

This study aims to establish that plasma treatment of aqueous dairy manure, hereafter referred to as animal waste, will (1) suppress ammonia volatilization by reducing the pH of animal waste and (2) enrich animal waste in total nitrogen due to the retention of nitrogen in ammonia and the addition of nitrite and nitrate (i.e., nitrogen oxyanions). This is complex in that both plasma-biowaste chemistry and microbial interactions within biowaste have confounding effects on plasma treatment outcomes. Here, our investigation is focused on plasma-biowaste chemistry only, excluding the role of microbial interactions. Of particular interest is to determine whether compounds in a biowaste slurry might interfere with acidification, or nitrogen oxyanion generation, via air-plasma treatment. For example, the biowaste compounds might react with the plasma-generated species (e.g., NO_*x*_) and prevent reactions in eqs 7–10 from occurring. Moreover, other possible undesirable side reactions, such as the oxidation of ammonia occurring as a result of plasma treatment, may influence the nitrogen content. While recent studies have reported nitrogen oxyacid generation in water via plasma treatment,^19,30−33^ the extent of nitrogen oxyanion enrichment in biowaste and its downstream effects on biowaste acidification remain unestablished. Here, we demonstrate that treating animal waste with air-plasma effluent containing NO_*x*_ reduces its pH, enhances the overall nitrogen content and, thus, upcycles the waste, and reduces the major health and environmental hazards posed by ammonia volatilization. We also study the yield of nitrogen oxyanions from plasma-generated NO_*x*_ species. Whether plasma treatment can add an adequate amount of nitrogen oxyanions to animal waste or whether it removes ammoniacal nitrogen from waste will have important implications for the design and optimization of sustainable LTP-assisted processes for green fertilizer manufacture on farmlands. Furthermore, various species of nitrogen oxyanions, mainly nitrite and nitrate, are subject to different regulatory requirements in agricultural applications, which necessitates a better understanding of their formation via plasma treatment.

## Methods

### Experimental Setup

**Figure 1:**
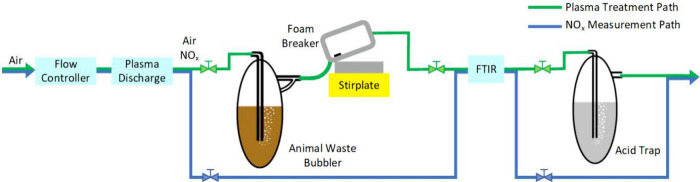
Schematic of the custom-built experimental setup for the plasma treatment of animal waste. NO_*x*_ is generated by using a dc glow discharge in air at a steady-state concentration of 5000 ppm. Before treatment of animal waste, a NO_*x*_-rich stream is routed around the animal waste bubbler to the FTIR instrument to measure the steady-state concentration of NO_*x*_ in the air-plasma effluent (blue path). Animal waste samples, placed in the animal waste bubbler, are treated with the air-plasma effluent, while ammonia is absorbed from the effluent into the acid trap (green path). A stirred foam breaker is used to reduce foaming of dairy manure so air or plasma effluent can separate from the animal waste.

Figure 1 shows a schematic of the setup built for the air-plasma treatment of animal waste, which consists of several components. In the blue path, air flows through the air-plasma discharge, which creates NO_*x*_. The NO_*x*_-rich effluent is routed to the Fourier transform infrared (FTIR; Bruker Vertex 70) instrument to measure the NO_*x*_ concentration at steady state. In the green path, air is routed through the air-plasma discharge; this plasma effluent is NO_*x*_-rich. The plasma effluent is bubbled through a bubbler filled with samples of animal waste (brown in Figure 1). This contact absorbs NO_*x*_ from the plasma effluent into the animal waste. The plasma effluent also strips ammonia from the animal waste into the effluent stream. The animal waste mixture foams aggressively; thus, the plasma effluent and animal waste foam are separated in a foam breaker. The effluent is then directed to the FTIR instrument, where the remaining NO_*x*_ is measured. The effluent continues downstream to the acid trap (green in Figure 1), where ammonia in the plasma effluent is absorbed.

**Figure 2:**
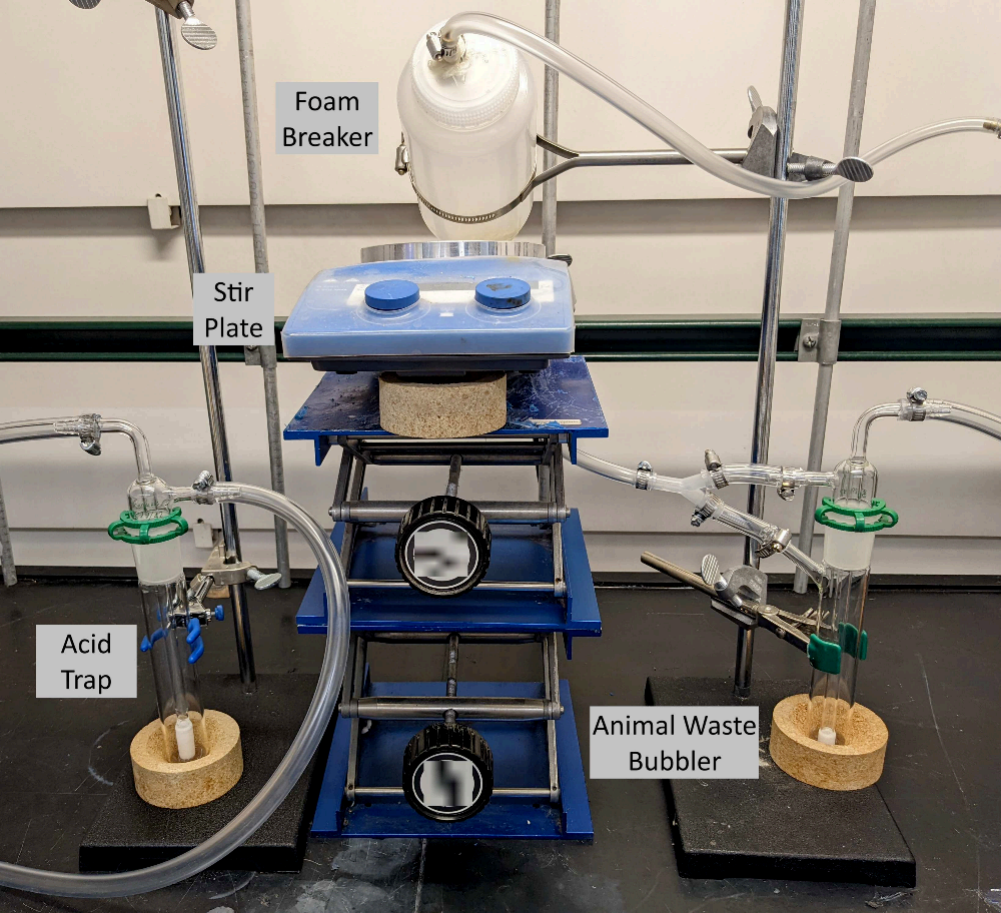
Custom-built experimental setup for air-plasma treatment of animal waste. The animal waste bubbler, foam breaker, and acid trap are depicted.

The physical construction of the foam breaker, animal waste bubbler, and acid trap can be seen in Figure 2. The animal waste bubbler is the component in which the plasma effluent or air is bubbled through the animal waste samples. The bubbler is a modified borosilicate vacuum trap (Chemglass CG-4516-01). The internal chamber had a diameter of 2.8 cm and a height of approximately 23 cm. The internal tube of the bubbler is replaced with a gas dispersion tube (Chemglass CG-203-03). A glass dispersion tube is a hollow tube with a coarse frit at the end. Gas enters the entry port (top of the bubbler) and passes through the internal dispersion tube to emerge from the frit and form small bubbles when the tube is immersed in aqueous samples. The frit is cut to 1.4 cm and touches the bottom of the bubbler body. The gas dispersion tube is cut to 22.5 cm and has an outer diameter of 8 mm. The entry and exit ports of the bubbler (exiting the sides of the CG-4516-01 trap) are replaced with hose barbs that have an internal diameter starting at 10 mm and tapering to approximately 4 mm. These enable the easy attachment of polyethylene tubing. A second exit port is added to the CG-4516-01 trap, a 6 cm long borosilicate tube with a 6.3 mm inner diameter that is joined to the bubbler body at 0.63 rad. This second exit port improves drainage from the foam breaker to the animal waste bubbler. The two exit ports connect to a plastic wye (shaped like the letter “Y”), which connects to the foam breaker. All connections are secured with hose clamps. The acid trap bubbler is nearly identical with the animal waste bubbler with two differences. First, the frit on the gas dispersion tube is full size (4 cm), which touches the bottom of the bubbler body. Second, the acid trap bubbler does not have a second exit port.

The foam breaker is designed to reduce manure foaming so that vapor (i.e., air, or plasma effluent) can separate from the animal waste. The foam breaker is a 1 L polypropylene cylindrical bottle (Deschem Labware PPXKP1000MLGK). The body is 9 cm in diameter and 17 cm in height. The screw-on lid is 5.5 cm wide and 2 cm long. A 0.5 cm hole is etched into the rounded corner of the bottle, and polyethylene tubing is glued over the hole. One hole (0.63 cm) is etched on the lid to fit a barbed 0.63 cm brass metal tube through it. The seam between the brass and the lid is filled with liquid cement. The foam beaker has a stir bar placed on the side of it and is positioned atop a stir plate. It is tilted to encourage the animal waste to drain into the animal waste bubbler.

LTP is generated using a direct-current (dc) pin-to-pin glow discharge in air. The plasma discharge consists of two metal pins separated by 8 mm; one pin is connected to the ground and the other to a high-voltage power supply. These pins are encased in a borosilicate tube so that the plasma effluent is confined. Tubing is connected to each end of the pin-to-pin plasma discharge to enable air flow; see ref (33) for a detailed description of the discharge used in this work, which is operated at 6 kV (Spellman High Voltage, SL10PN1200) with an air flow rate of 1 slpm (standard liters per minute). Utility air is used, which has an atmospheric CO_2_ concentration and is dried to remove water. The air-plasma discharge generates approximately 5000 ppm of NO_*x*_ in its effluent.

### Experimental Procedure

Animal waste was collected as freshly deposited dairy manure at DeJaeger Farms in Merced, CA. Biowaste was vacuum-sealed into plastic bags and heated to 85 °C for 10 h to eliminate microbes and pathogens. This enabled better control over the manure slurry to only investigate the plasma-biowaste chemistry by eliminating the influence from the microbes. Biowaste was then frozen at −20 °C and stored before use. Prior to use, a 500 mL aliquot was homogenized (Nutribullet NB-WL088D-23) and then stored in jars to be frozen for later retrieval; it was then refrozen until retrieved for experiments.

Experimental samples are prepared the same day that they are used for plasma treatment. Samples are prepared by weighing 50 g of room temperature homogenized manure in a centrifuge tube with an analytical balance (Mettler Toledo MS1045) and depositing the animal waste into a 250 mL borosilicate three-necked flask. Mixed into the flask are 18.9 mL of 5% (w/v) NH_4_Cl in water and 81 mL of distilled water. The pH is approximately 7.4 at this point. NaOH (1 M), typically 4.6 mL, is added until the pH reaches 9. As the biowaste samples are pasteurized, no microbes are present to hydrolyze the urea to ammonia. Normally, urea is hydrolyzed by urease to form ammonia and carbamic acid. The resulting carbamic acid is unstable and reacts very rapidly with water to form carbonic acid and ammonia, for a net production of two ammonia molecules per urea.^34^ As a result of this sterilization, the manure samples are ammonia-deficient prior to the addition of NH_4_Cl. This is an issue because it is desirable to observe the impact of air-plasma treatment on ammonia volatilization from the manure, which is challenging if there is no ammonia. Ammonia volatilization is gauged by the ammonia content captured in the downstream acid trap (see Experimental Setup). The more un-ionized ammonia in the manure, the more ammonia that will be stripped into the air (per Henry’s Law) and finally deposited into the acid trap. It is desirable to ensure that there is enough un-ionized ammonia in the manure that there would be sufficient ammonia to measure in the downstream acid trap. Hence, NH_4_Cl and NaOH are added to the manure slurry, as above, to achieve measurable ammonia in the downstream acid trap for the case of no plasma treatment. Raising the pH increases the ratio of ammonia to ammonium. This pH level is within the typical range for manure.^15^ In addition, reference experiments are performed with biowaste-free samples that are handled identically, except that 50 g of distilled water is added in place of the biowaste slurry. All sample transfers are made via glass pipettes. The manure slurry is mixed via a stir bar during preparation. Aliquots of 30 mL are pipetted into the animal waste bubbler in Figure 1. The acid trap is used to capture ammonia that might be stripped into the gas phase out of the manure slurry. The acid is a 30 mL aqueous solution of 0.04 N HCl for the capture of ammonia, inspired by the method 4500-NH_3_ B of the *Standard Methods for the Examination of Waste and Wastewater*.^35^

The procedure for the air-plasma treatment of manure samples is as follows. The air-plasma discharge is activated and allowed to come to steady state, following the blue path in Figure 1. The concentrations of NO_*x*_ and HONO are measured by FTIR. Then, the flow path is switched from blue to green. This bubbles NO_*x*_-rich effluent from the air-plasma discharge through the animal waste bubbler and the acid trap, termed plasma treatment. After the sample is plasma-treated for 0, 2, or 6 min, the plasma discharge is deactivated. Subsequently, the air flow continues for 60, 58, or 54 min, for a total bubbling time of 1 h. The FTIR instrument measures the concentrations of NO_*x*_ and HONO during this time. After plasma treatment and air bubbling for 1 h, the pH and mass of the treated samples are measured. Ammonium in the acid trap is also measured; captured ammonia is ionized to ammonium in the acid trap. The samples are then refrozen to slow chemical transformation and preserve them for later nitrogen analysis, as described below.

### Measurement Techniques

Ammonium in the acid trap is measured using an ion-selective electrode (ISE; Hanna Instruments HI- 4100). For calibration and sample analysis measurements, 30 mL aliquots at room temperature (22 °C) are placed within 50 mL borosilicate three-necked flasks, and the pH is raised above 11 by adding 0.6 mL of an ionic-strength adjustment solution (HI 4001-00). A calibration curve for the ISE is constructed by using solutions of 1000 ppm (HI 4001-03) and 100 ppm (HI 4001-02) and diluting them as necessary. Moreover, pH measurement is made by HI 1083P for all measurements, except for samples in batches B5–B8 (Table 1) and the destruction tests, for which HI 1053B is used. pH readings are made using HI 3022.

**Table 1:** Conditions of Air-Plasma Treatment in Different Batches of Prepared Samples^a^

batch name	untreated (0 min plasma; 0 min air)	0 min (0 min plasma; 60 min air)	2 min (2 min plasma; 58 min air)	6 min (6 min plasma; 54 min air)	HNO_3_ added (0 min plasma; 60 min air)
B0		1		1	
B1		1	1	1	
B2		1	1	1	
B3		1	1	1	
B4		1	1	1	
B5					3
B6	3				
B7	1				
B8	1	2		2	

a Samples are first bubbled with air-plasma effluent, where applicable, and then bubbled with air, as depicted in Figure 1. For each batch, the table summarizes the number of samples in each batch that underwent the indicated treatment conditions. For example, three samples were drawn from batch B2; one was treated at “0 min”, one at “2 min”, and one at “6 min” conditions. “HNO_3_ added” samples serve as control samples, wherein HNO_3_ is directly added to animal waste in a quantity identical with the amount of nitrogen absorbed by “6 min” samples, followed by bubbling with air for 60 min. Samples in batch B8 contain no animal waste and are denoted by “no biowaste”.

The concentrations of nitrite and nitrate in the treated samples are measured by ion chromatography (IC) using an IonPac AS-11 anion-exchange column from ThermoFisher. The column is protected by an upstream IonPac AG-11 guard column. Anion chromatography is measured with a Dionex ICS 3000 chromatograph with a conductivity detector. Flow rates are at 1.0 mL/min. The system temperature is ambient. The eluent ranges from 5 to 36 mM NaOH. The eluent follows a gradient; it starts at 0.5 mM for 2.5 min, increases from 0.5 to 5 mM over 3.5 min, and then increases from 5 to 36 mM over 12 min. Samples are diluted and then prepared for analysis by filtering them through a 0.2 μm syringe filter (Corning 431215) into analysis vials. The vials are procured from Thermo Scientific (C5000-75C) with caps from Agilent (5182-0715). The samples for IC are prepared the day before analysis and refrigerated at 2 °C. The vials and caps are wrapped in parafilm to prevent volatilization. Sample recovery is approximately 100%. The measured concentration is linear with dilution.

**Figure 3:**
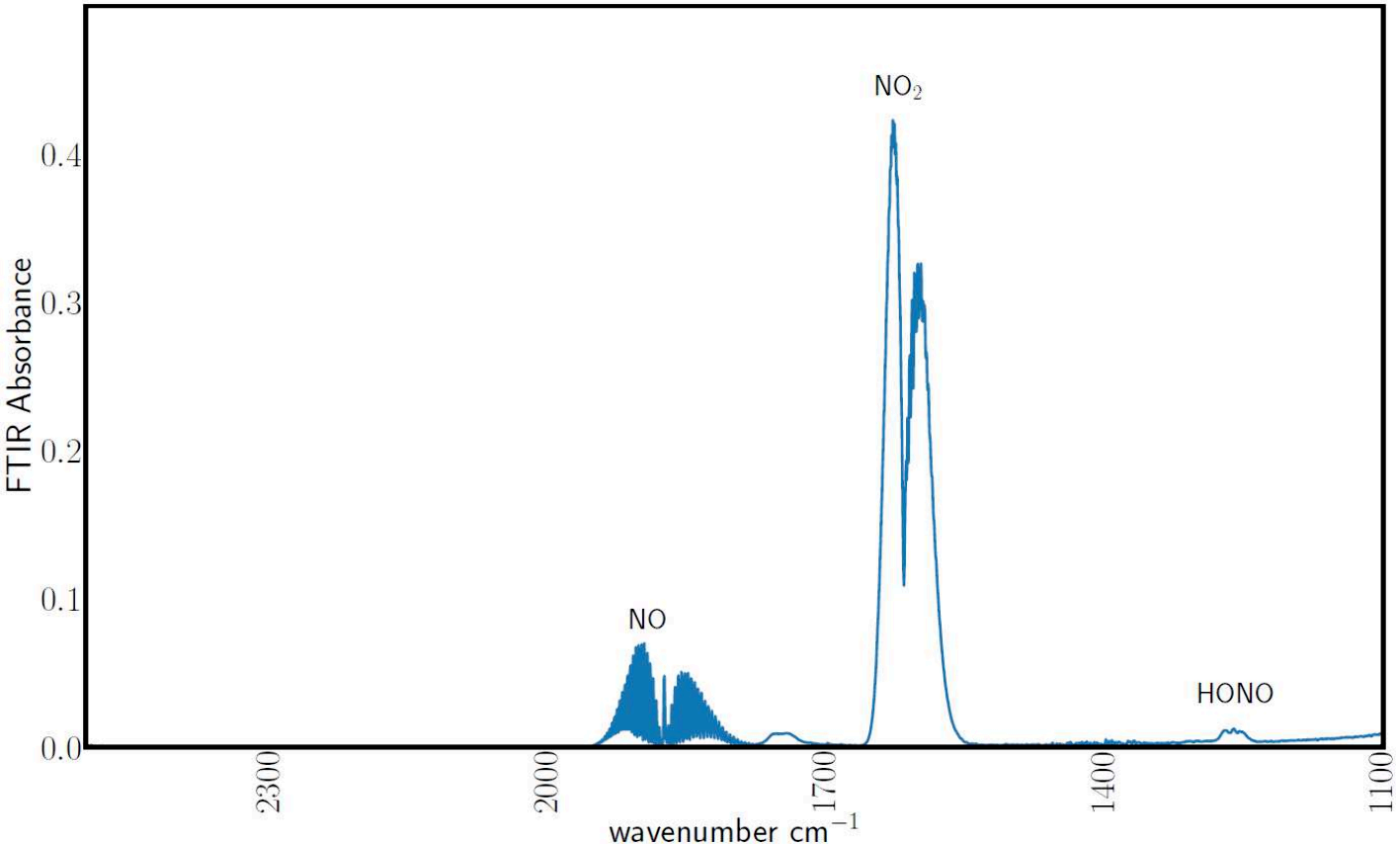
Absorbance spectrum of the air-plasma effluent at steady state, as measured by FTIR. The spectrum corresponds to approximately 3400 ppm of NO, 1670 ppm of NO_2_, and 110 ppm of HONO. NO is fit between 1801 and 1950 cm^–1^, NO_2_ is fit between 1550 and 1675 cm^–1^, and HONO is fit between 1201 and 1321 cm^–1^.

The NO_*x*_ concentration in the air-plasma effluent is measured using FTIR (Bruker Vertex 70) with a 10 cm glass gas cell (A-132). Scans are done with 1 cm^–1^ resolution. A total of 10 background scans are taken before each measurement, with a wavenumber range from 980 to 3800 cm^–1^. Scans report absorbance and use a mid-infrared source with KBr windows and a LN-MCT Mid VP detector. A representative spectrum is given in Figure 3. No ozone or N_2_O can be observed (expected wavenumbers 1100 and 2200 cm^–1^, respectively) in the collected FTIR spectra.^33,36^ The concentrations of NO_*x*_ and HONO are determined in a two-step procedure.^33^ In the first step, a simulated spectrum is fitted to a measured FTIR spectrum. The species concentrations are adjusted to find a best fit. Parameters for the simulated spectrum are taken from the HITRAN database.^37^ The simulated spectrum is generated with a computational step of 0.01 cm^–1^, an optical path of 20 cm, an assumed Gaussian apparatus function, and an apparatus resolution of 1 cm^–1^. In the second step, the calculated concentrations from the first step are adjusted using a calibration curve. The calibration curve is constructed by comparing values in step one to known concentrations of NO (Linde NI NO5000C-A3) and NO_2_ (Linde NI NX2000C-A3), added in various different ratios.

The total nitrogen (TN) and total Kjedlahl nitrogen (TKN) contents of the plasma-treated animal waste samples are measured. TKN is the sum of organically bound nitrogen and ammoniacal nitrogen but does not include azide, azine, hydrazone, nitrate, nitrite, nitrile, nitro, nitroso, oxime, or semicarbazone.^35^ On the other hand, TN is the sum of all nitrogen in the sample.^35^ TKN and TN are defined, respectively, as
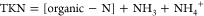
11
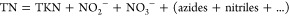
12The TKN and TN analyses were carried out by the Analytical Laboratory, University of California, Davis.^38,39^ They were found to have nearly 100% recovery in the spiked samples. The TKN is analyzed via Method 4500-N_org_ D of the *Standard Methods for the Examination of Water and Wastewater*(35) by digesting the sample using a Lachat model BD-46 block digestor such that the TKN is transformed into ammonia and then measured with a Timberline TL-2900 dual-channel ammonia analyzer. The TN is analyzed via the combustion method^40^ using a Leco TruMac carbon and nitrogen analyzer, where all nitrogen in a sample is converted to NO and then measured.^39^

In summary, the NO_*x*_ species generated in the air-plasma discharge can be absorbed into the manure samples and are expected to form nitrogen oxyanions. It is possible that the NO_*x*_ species, or the reactive nitrogen oxyanion products, could react to form other species. To track the fate of nitrogen, the amount of nitrogen absorbed from the plasma discharge is measured by FTIR. The quantity of nitrogen oxyanion generated by the plasma in the manure samples is measured by IC. Ammonia in the manure slurry can be retained in the manure, can react, or can evaporate. To account for ammonia and other organic nitrogen, the TKN is measured. Ammonia volatilization is also gauged by measuring the ammonia content in the acid trap. Nitrogenous side products in the manure can be accounted for by measuring the TKN and TN.

## Results and Discussion

### Reduction in Ammonia Volatilization

**Figure 4:**
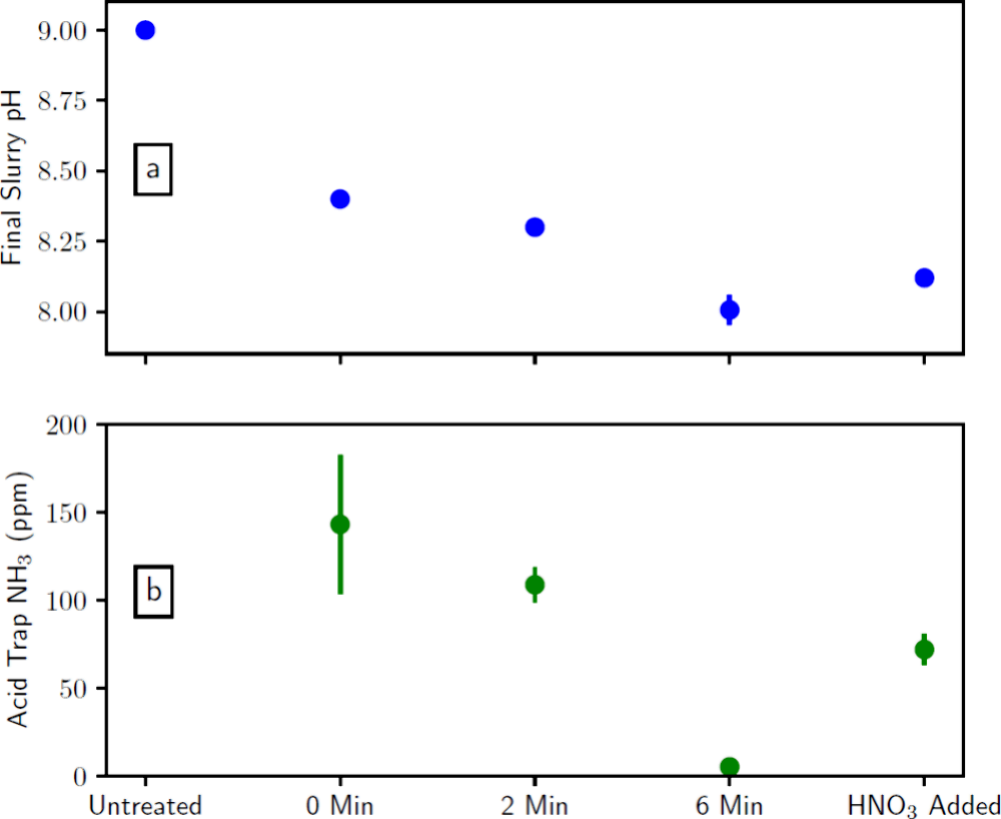
Air-plasma treatment of animal waste in batches B0–B7. (a) Final pH of the aqueous slurry. (b) Concentration of NH_4_^+^ measured in the acid trap following each treatment. The bars represent 1 standard deviation about the mean for an average of three samples.

The treatment conditions for different experimental batches are listed in Table 1. Figure 4 shows the impact of air-plasma treatment on (a) the pH of an aqueous slurry and (b) the amount of ammonia absorbed from the gas phase into the downstream acid trap in batches B0–B7.† The “0 min” samples have reduced pH compared to the untreated samples, likely due to absorbing CO_2_ during the 60 min bubbling period. The slurry pH drops with increasing air-plasma treatment time, as shown previously for the plasma treatment of water.^41^ Note that air is bubbled for a full hour in all samples except for the “untreated” samples, which can result in acidification from carbonic acid formed from CO_2_ absorbed from air.^42^ The influence of CO_2_ versus acidification from plasma can be disentangled by comparing the “0 min” samples to the plasma-treated samples (i.e., “2 min” and “6 min” samples). This is because the “0 min” samples are only exposed to CO_2_ from air, whereas the plasma-treated samples have additional acidification from the plasma-generated nitrogen oxyacids. Plasma treatment also results in a decrease in the concentration of ammonium measured in the acid trap. These observations suggest that air-plasma treatment acidifies animal waste as in eqs 2–10 and, hence, reduces ammonia volatilization.

**Figure 5:**
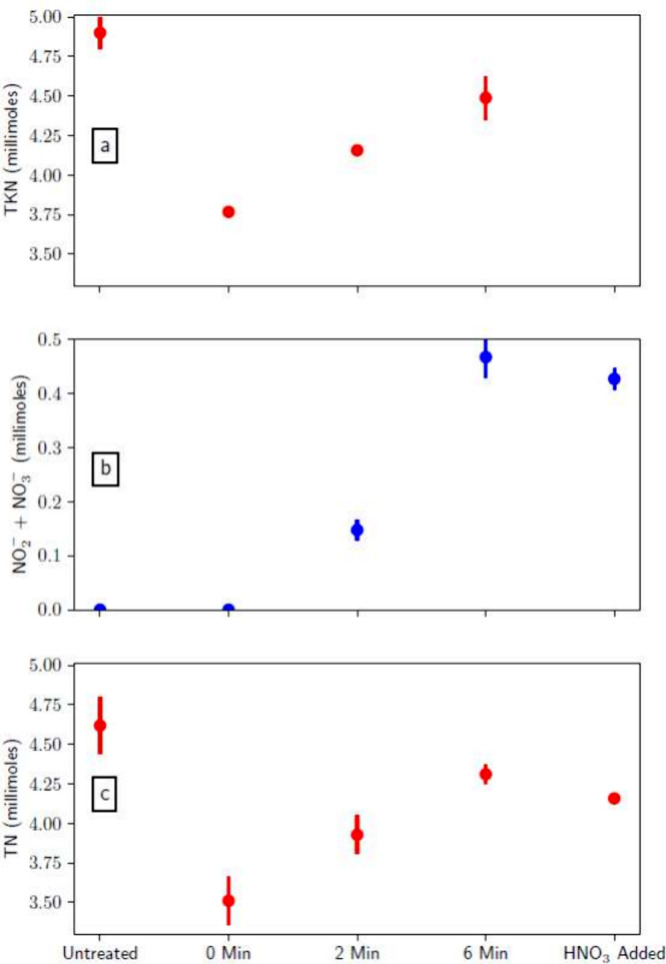
Nitrogen content of animal waste following air-plasma treatments in batches B0–B7. (a) Total Kjeldahl nitrogen (TKN) measured in batches B0 and B1. Note that there is no entry for “HNO_3_ added” samples because they correspond to batch B5. (b) Combined nitrite and nitrate concentrations measured in batches B2–B5. (c) Total nitrogen (TN) measured in batches B2–B5. All “untreated” samples are drawn from batches B6 and B7. The points in subplot a represent the average of two samples. The points in subplots b and c represent the average of three samples. The bars represent 1 standard deviation of the mean.

**Figure 6:**
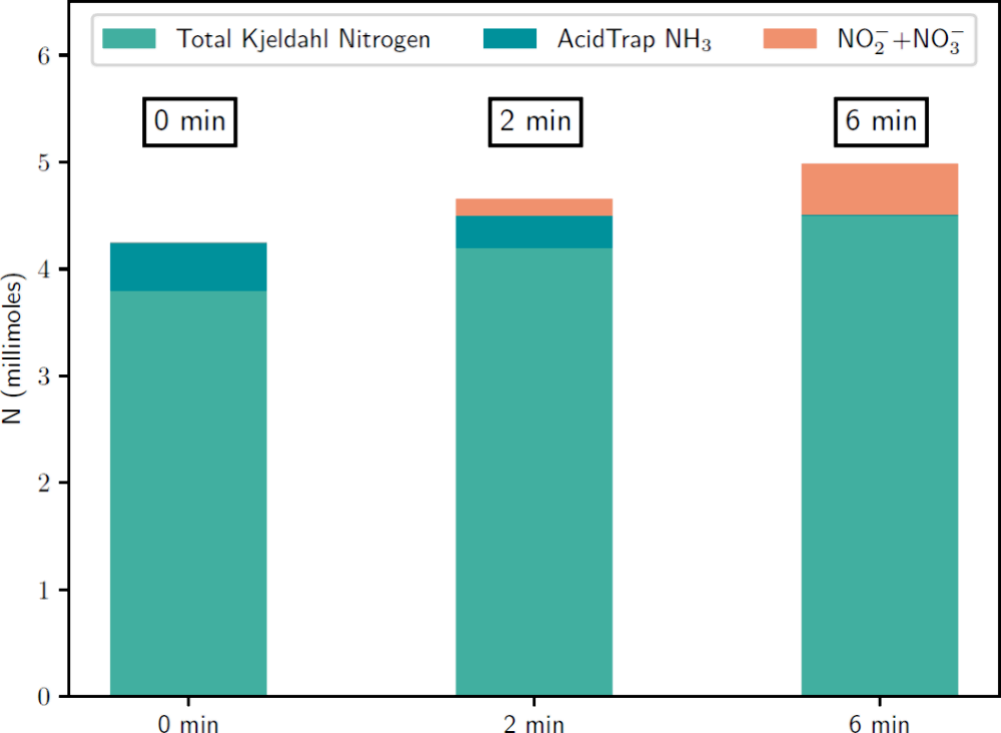
Average final distribution of nitrogen within the system (manure slurry and acid trap) for the “0 min”, “2 min”, and “6 min” samples (B2–B4). The TKN and nitrogen oxyanion content of the manure slurry, as well as the acid trap ammonia content, are shown.

**Figure 7:**
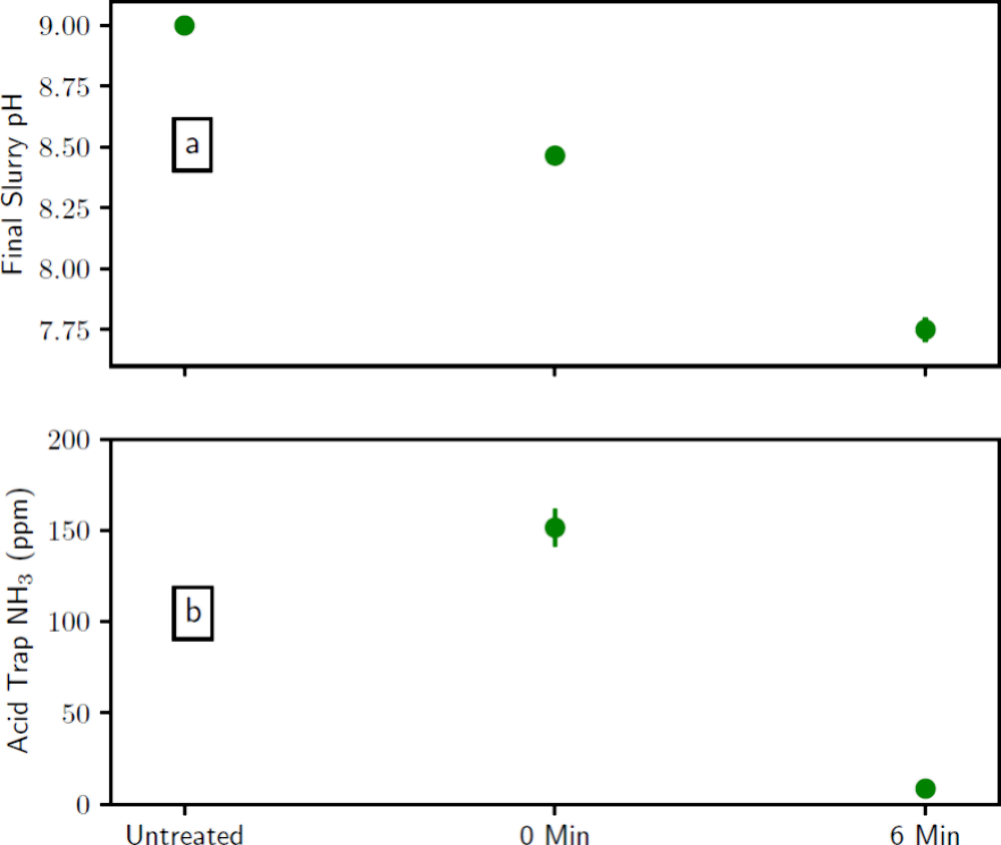
Air-plasma treatment of NH_4_Cl solutions in batch B8 for treatments of 0 min and 6 min. (a) Final pH of the mixture. (b) Concentration of NH_4_^+^ measured in the acid trap following each treatment. The bars represent 1 standard deviation about the mean.

Figure 5 shows (a) the measured TKN, (b) the combined nitrite and nitrate concentrations, and (c) the measured TN in batches B0–B7. It is observed that reduced ammonia volatilization leads to an increase in the ammonia/ammonium content of the animal waste mixture, as reflected by the increase in the TKN content of the samples (see eq 11). Hence, acidification of the solution results in retaining ammonia as ammonium, which can, in turn, reduce the loss of ammoniacal nitrogen. The distribution of nitrogen in the treated manure slurries (B2–B4) and the acid traps can be seen in Figure 6. A reference experiment sheds light on the role of the manure slurry in this work. Samples in B8 were prepared identically with the other batches, but with distilled water in place of manure. Figure 7 shows the effect of air-plasma treatment on the manure-free samples for both (a) solution pH and (b) NH_3_ acid trap content. Air-plasma treatment reduces the solution pH and reduces the NH_3_ acid trap content. This follows the trend shown in Figure 4. The TKN, nitrogen oxyanion content, and TN follow the same trends as those in Figure 5, but they are not shown. These results suggest that the biowaste compounds do not alter the expected chemistry of nitrogen oxyanion generation and ammonia evaporation.

**Figure 8:**
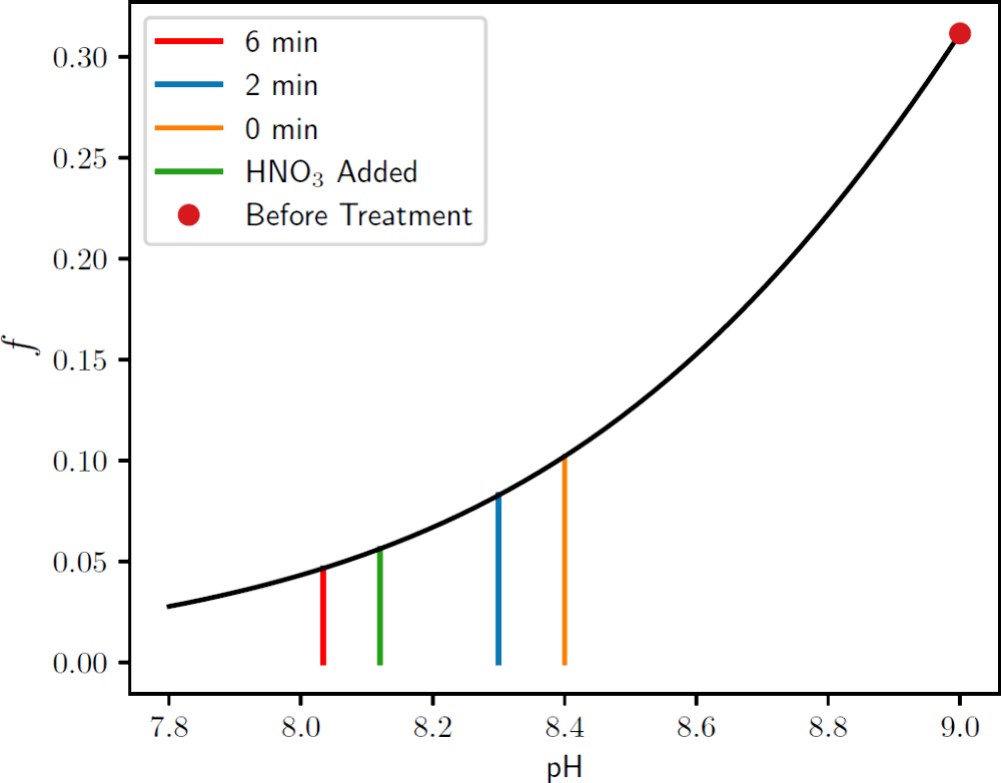
Fraction of un-ionized ammoniacal nitrogen, , as a function of pH changes of animal waste samples in batches B0–B7. The black curve shows the theoretical equilibrium value for *f*, derived from the Henderson-Hasselbalch equation^43^ for the reaction in eq 1. The colored vertical lines represent the average pH of the samples for air-plasma treatment times of 0, 2, and 6 min, as well as “HNO_3_ added”. The intersection of vertical lines with the black curve indicates the degree of ionization of ammonia in these samples. Note that “6 min” of plasma treatment leads to a final *f* that is almost half as large as the *f* value associated with “0 min” of plasma treatment (i.e., only 60 min of air bubbling).

That small variations in the pH can lead to significant differences in ammonia capture in the acid trap and, thus, ammonia volatilization from the slurry is explained by recalling that only ammonia is volatile, not ammonium. The reversible reaction between ammonium and ammonia (eq 1) is pH-dependent, as can be described by the Henderson-Hasselbach equation.^43^ Here, all sample treatments start at pH 9, with approximately one-third of their ammoniacal nitrogen in the form of NH_3_. According to Henry’s Law,^44^ the equilibrium partial pressure of NH_3_ in the air stream in contact with the slurry increases proportionally with the concentration of NH_3_ in the slurry. The early acidification due to air-plasma treatment is expected to lower the pH and, hence, the ammonia available for volatilization. Figure 8 shows the theoretical fraction of un-ionized ammoniacal nitrogen, defined as
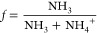
13in relation to the pH changes of the animal waste samples in batches B0–B7. This figure suggests that a small drop in the pH of even 0.1 or 0.2 units from pH 9 can significantly depress ammonia volatilization. The samples with 6 min of air-plasma treatment (i.e., 54 min of air bubbling) have a post-treatment pH of approximately 8.0 with *f* = 0.046. By comparison, the samples with 0 min of air-plasma treatment (i.e., 60 min of air bubbling) have a post-treatment pH of 8.4 with *f* = 0.098, which is more than twice the fraction of un-ionized ammoniacal nitrogen compared to the “6 min” samples. As such, we observe that small pH changes can significantly impact the quantity of ammonia available for volatilization because the pH range of interest (pH 8–9) is in the neighborhood of the p*K*_a_ value of ammonia. Conceivably, early pH depression can drop the quantity of ammonia evaporated over the course of 1 h. It is worth noting that other manure samples may behave differently in terms of the initial pH, or buffering capacity. This is because the manure pH and its chemical content varies over time, with the generation of ammonia from urea, as well as by animal feed, species of animal (and between animals of the same species), how the manure is processed, and manure additives (for example, if animal bedding is swept into the manure).^13,15,45−49^

### Upcycling of Animal Waste

From the above results, we also observe that air-plasma treatment enriches the nitrogen content of animal waste. This occurs due to both retaining nitrogen in ammonia and adding plasma-generated nitrite and nitrate species to the animal waste slurry. Figure 5 shows that samples exposed to air-plasma effluent have increased TKN levels, as well as greater nitrogen oxyanion content, compared to the “0 min” samples. Most importantly, the plasma-treated samples show greater TN levels. Note that samples treated for “6 min” have less TN and TKN than the original “untreated” samples. This can be attributed to the loss of TKN (as ammonia) during the air bubbling step, as is evident for the “0 min” samples. Air-plasma treatment enhances the TN and TKN content of animal waste relative to the “0 min” samples because plasma treatment reduces evaporative ammonia loss and adds exogenous nitrogen. However, while the TKN rises, it is possible that the ammonia in the TKN is transformed to other forms, as discussed next.

### Ammonia Transformation or Retention?

It is worth determining whether the reduction of ammonia seen in the acid trap in Figure 8 with increasing plasma treatment could be due to complete oxidation of ammonia to dinitrogen. Alternatively, it is possible that ammonia in the animal waste is transformed to some species that is still measured in TN or TKN. Air-plasmas have been known to generate reactive oxygen and nitrogen species (RONS), including hydroxyl radicals, hydrogen peroxide, ozone, NO_*x*_, HONO, and more. These are all potent oxidizers that could react with ammonia. Discussions of these species, and other reactions and products, are provided elsewhere.^50−56^

It is unlikely that ammonia in the animal waste is being completely oxidized to dinitrogen by plasma. Note that dinitrogen is extremely volatile and would be lost from the sample because it is nearly insoluble in water. Further, dinitrogen is not detected by the TKN. Because the TKN rises with treatment (Figure 5), it can be inferred that the rise in the TKN with air-plasma exposure is either ammonia-retained or ammonia-transformed to some organic nitrogen-containing compound that is included in the TKN (see eq 11). If complete oxidation of ammonia to dinitrogen occurs in the biowaste, the effect will be small relative to the retention of nitrogen as the TKN because the TKN increases with plasma treatment.

**Figure 9:**
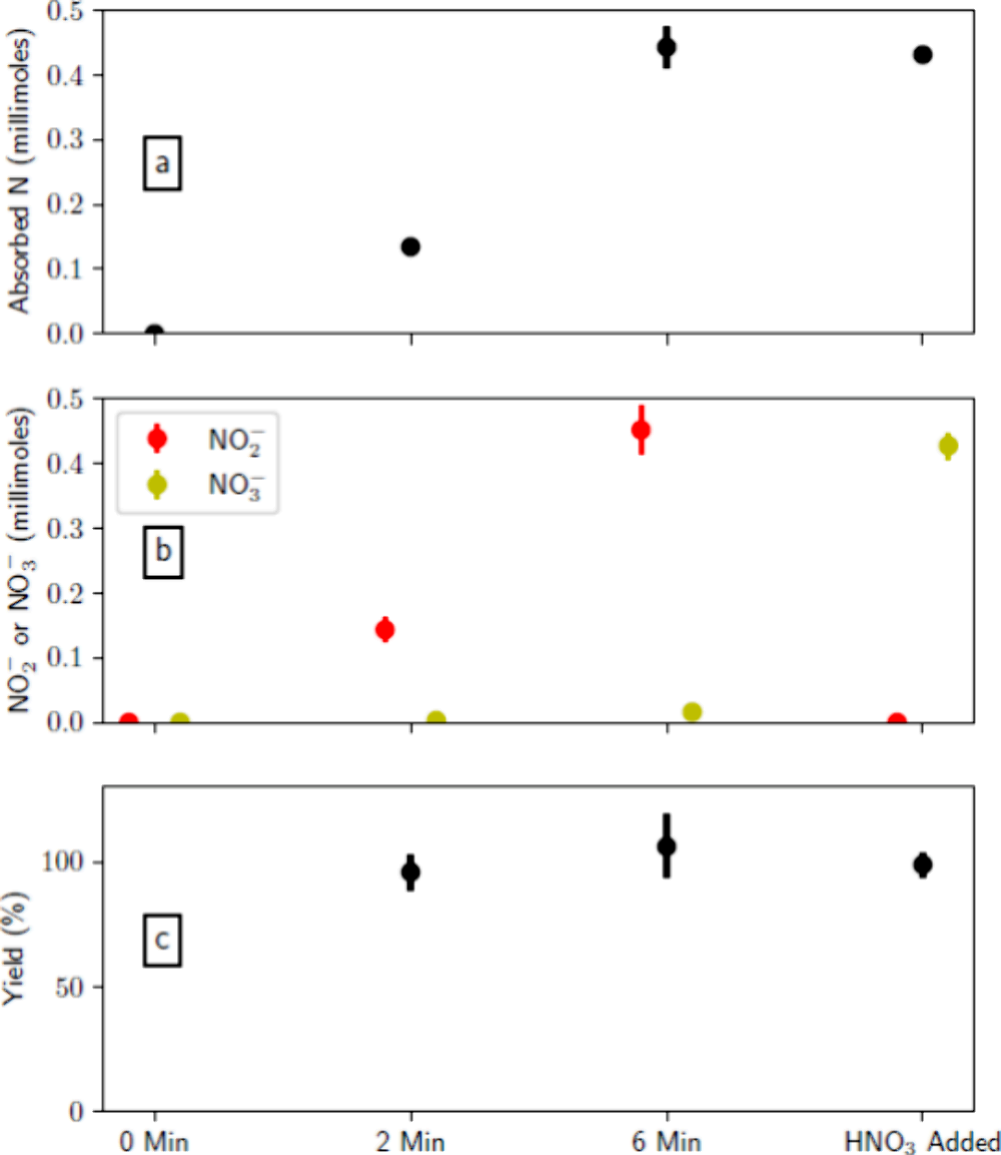
Fate of plasma-generated nitrogen as absorbed by animal waste in batches B2–B5. (a) Millimoles of nitrogen absorbed from the air-plasma stream as measured by FTIR, or added exogenously in the case of “HNO_3_ added”. (b) Millimoles of nitrite or nitrate species in the animal waste samples. (c) Yield of nitrogen oxyanion from nitrogen absorbed. The points represent the average of all three samples, whereas the bars represent 1 standard deviation of the mean.

If RONS were destroying ammonia, then it is worth considering some of the common species involved. It is unlikely to be ozone here because ozone production is aggressively quenched by small quantities of NO_*x*_^57^ and there is no obvious peak in the FTIR spectrum of the air-plasma effluent that would represent ozone in Figure 3, which would occur near 1100 cm^–1^.^36^ While it is possible that nitrite and nitrate can oxidize ammonia,^28^ or other absorbed nitrogen species, this is considered unlikely because Figure 9 demonstrates that all exogenous nitrogen (either added nitric acid in B5, or absorbed from air-plasma effluent in B2–B4) remained as nitrite and nitrate at the time of IC analysis and, hence, could not have reacted several months after the samples were generated. Air-plasmas are also known to form hydroxyl radicals and hydrogen peroxide (formed from hydroxyl radical coupling), which are potent oxidizers.^19^ Formation of these species is due to the water present in the air-plasma discharge, usually from air moisture. Even though the utility air used in this work is dried, there exists some low quantity of moisture, which provides the hydrogen in HONO. Hydroxyl radical has a lifetime of less than 1 s in atmospheric air,^58^ which is much less than the approximately 4 s transport time from the plasma discharge to the manure in our experiments. The time of 4 s was arrived at by noting that the length of the tube from the discharge to the manure was over 30 feet in length and had an internal diameter of 3 mm and the flow rate was 1 slpm. It has been argued that the bulk of the hydroxyl radicals produced are generated in the gas-phase discharge and not at an aqueous surface, so it is not inconceivable to have hydrogen peroxide in this discharge, which could act to consume ammonia or ammonium.^52^ The FTIR spectrum of hydrogen peroxide overlaps with HONO, although Figure 3 lacks the characteristic peak seen in the spectrum from this discharge.^59^ If there is hydrogen peroxide, then the quantity will be nearly negligible, and it is unlikely that it can explain the suppression of ammonia in the acid trap. Atomic oxygen also has a lifetime of less than 1 s,^60^ and so it is not likely to react with the manure to any significant extent.

It is also instructive to compare the plasma-treated samples to a control. Our central premise is that the NO_*x*_ in air-plasma would make nitrogen oxyacids, which would reduce the pH of the solution and, in so doing, reduce ammonia volatilization. A good standard of comparison would be to compare plasma-treated samples to samples that are not plasma-treated, but are still acidified and enriched with nitrogen oxyanion. This would enable us to isolate how much plasma treatment differs from acidifcation alone. The proposed untreated samples can be made by preparing a batch containing nitric acid, with the molar nitrogen content equivalent to the amount of nitrogen oxyanion absorbed by samples (B2–B4). Here, B5 serves as this control. If acidification by nitrogen oxyacids is the dominant mechanism, then B5 should have approximately the same properties as the “6 min” samples in B2–B4 (i.e., the pH, nitrogen oxyanion content, TN, and ammonia content in the acid trap). B5 is approximately the same in all of these metrics. Further, it is unlikely that the nitrogen oxyanions could have reacted and caused some change in ammonia because the exogenous nitrogen is entirely accounted for as nitrite and nitrate, and not some other form (Figure 9). The two systems were composed of the same material, with the only difference being the route of nitrogen exposure. Yet, they exhibit similar trends for pH, nitrogen oxyanion content, ammonium acid trap content, and TN. It is unlikely that there exists any mechanism in common beyond the reduction in the pH and reduced ammonia volatilization. However, this observation does not rule out that the air-plasma effluent might be converting ammoniacal nitrogen to a different form of the TKN, as noted above.

The B5 samples (“HNO_3_ added”) have approximately the same amount of nitrogen added as the absorbed nitrogen in “6 min” samples in B2–B4 and are, thus, an interesting comparison. A few discrepancies between the B5 samples and the samples in B2–B4 should be discussed. First, the nitrogen oxyanions of B5 are entirely nitrate, but the nitrogen oxyanions of B2–B4 are primarily nitrite (Figure 9b). In both cases, these ions scarcely react and, hence, are spectator ions to any possible reactions. Therefore, B5 is still a valid comparison to B2–B4 on the subject of acidification. Second, B2–B4 have slightly lower pH than B5 (Figure 4a), less ammonia in the acid trap (Figure 4b), and slightly more TN (Figure 5c). These are small differences. Note that B2–B4 have more nitrogen oxyanions and, hence, lower pH and more TKN, as expected. If the plasma effluent was oxidizing ammonia to dinitrogen, or otherwise removing nitrogen from the solution, the TN and TKN of B2–B4 would be less than those of B5, which is not the case.

**Figure 10:**
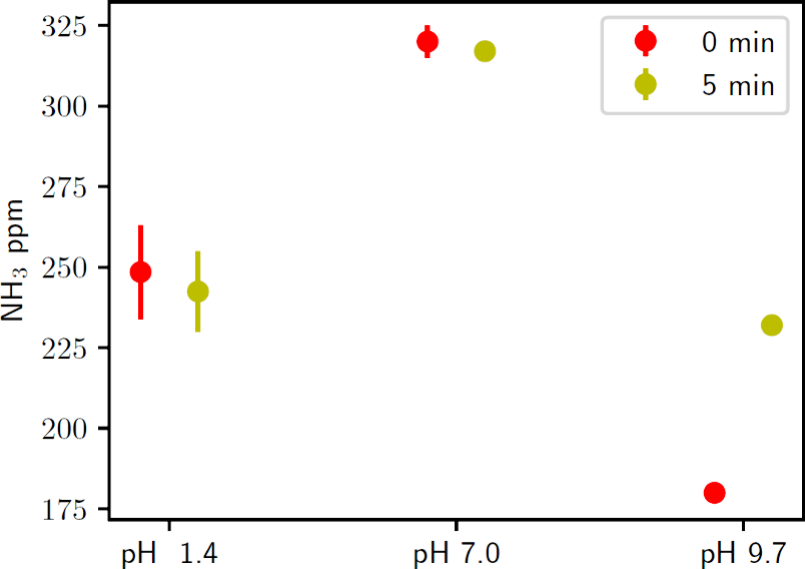
Ammonia content of different pH solutions of NH_4_Cl at 335 ppm following treatment with air-plasma for 5 min and bubbling with air for a total of 1 h. Control samples with only air bubbling show a similar ammonia content.

To further test the possibility of ammonia transformation or ammonium destruction in the acid trap, an acid trap with 335 ppm of NH_4_Cl and 0.04 N HCl was treated for 5 min under the same plasma conditions as the animal waste. A concentration of 335 ppm was selected because this is near, yet above the observed acid trap ammonia concentration measured in Figure 4. This was compared to “0 min” of air-plasma effluent exposure, with both having a total air bubbling time of 1 h. There is a negligible difference in the ammonia content between the treated versus untreated samples at pH 1.4 and 7 (Figure 10). The samples treated at pH 9.7 show more ammonia than the untreated pH 9.7 samples, likely due to the acidification-based retention from air-plasma treatment. The pH 7 and 9.7 samples bracket the range of the experiments for B0–B5, suggesting that plasma-based destruction of ammonia in the acid trap in this pH range is negligible compared to evaporative losses. All experiments were performed twice, and the results support the conclusion that the lower measurement of ammonia with air-plasma treatment is due to ammonia ionization by pH depression.

### Fate of Absorbed Nitrogen

The approximately 100% conversion of absorbed nitrogen to nitrite and nitrate is particularly interesting and is a promising sign for future engineering of these systems. While it is possible to contact NO_*x*_ with water to make acid and then blend that with manure to acidify it, directly acidifying manure slurry will reduce the need for a blending system. Further, minimizing water requirements reduces demands on agricultural operations. It is interesting to note that nitrite almost dominates the produced nitrogen in the slurry. It is not clear why this would be so, especially because nitrate is considered to be more stable.^27^ In the chemical reactions in eqs 2–10, eq 7 may be dominant. This is possible from a stoichiometric standpoint because there was approximately twice as much NO as NO_2_ in the inlet stream and approximately twice as much NO as NO_2_ in the outlet stream. Both species are very reactive.^61,62^ It could be that the NO_*x*_ species react quickly in a thin film at the liquid surface in accordance with eq 7, as suggested previously.^22^ This transport limitation would further reduce the likelihood to react with other species to form compounds like nitrosamines.^62^ Compared to previous reports, it is notable that there is no hydrogen peroxide or ozone observed in the air–plasma effluent in this work; hydrogen peroxide could oxidize nitrite to nitrate,^19^ which can explain the lack of nitrate. In general, the plasma discharge conditions can have a significant impact on the ratio of nitrite to nitrate by influencing the NO_*x*_ ratio,^33^ generating oxidizers like ozone,^63^ or producing hydrogen peroxide in a moist discharge.^19^

## Conclusions and Future Work

This work demonstrated that air-plasma treatment of aqueous dairy manure can mitigate ammonia volatilization, an important health and environmental hazard, and upcycle animal waste into a richer source of nitrogen. It was shown that plasma treatment lowers the pH of aqueous animal waste, which, in turn, enables retention of ammonia in the slurry in the form of ammonium. In addition, the TKN and TN contents of animal waste increase as a result of plasma treatment due to the retention of ammonia nitrogen and the addition of plasma-generated nitrogen oxyanion species. Adding nitric acid to the animal waste slurry confirmed that acidification is the primary mechanism responsible for ammonia retention. Future work will focus on investigating the energy efficiency of plasma-based nitrogen fixation, which is considered to be key for technology adoption.^12,33,64^ Future work will also include investigating plasma-treatment strategies that would shift the nitrogen oxyanion content away from nitrite toward nitrate and facilitate maintaining the nitrogen content of upcycled animal waste over time. Other important investigations include establishing how the native microbes in animal waste would affect the distribution of nitrogenous species in the biowaste following plasma treatment and how plasma treatment might change other biowaste components and micronutrients of interest to agriculture.

## Footnotes

† The calibration obtained for measuring the ammonia concentration in the acid trap is only valid down to 33 ppm; values reported below this range are by extrapolation.
